# Feeding injury of major lepidopteran soybean pests in South America

**DOI:** 10.1371/journal.pone.0271084

**Published:** 2022-12-15

**Authors:** Pablo Daniel Carpane, Matías Llebaria, Ana Flavia Nascimento, Lucía Vivan

**Affiliations:** 1 Bayer CropScience, Fontezuela, Buenos Aires, Argentina; 2 Universidade Federal de Uberlândia, Uberlandia, MG, Brazil; 3 Fundação Mato Grosso, Rondonópolis, MT, Brazil; University of Tennessee, UNITED STATES

## Abstract

Lepidopteran pests are major factors limiting soybean productivity in South America. In some cases, effective management of these species requires the use of foliar insecticides. For sustainable use of these insecticides, they should only be applied when insect population size exceeds an economic threshold. Since this estimation requires to determine the consumption of different species, this work aimed to integrate all these factors, studying the consumption of small (less than 1 cm long) and medium (1 to 1.5 cm long) size larvae of major lepidopteran pests to vegetative and reproductive tissues on Bt (M7739IPRO variety, containing the event MON87701 which expresses the Cry1Ac protein from *Bacillus thuringiensis*) and non-Bt (BMX Desafio RR variety) soybeans. The feeding injury to vegetative tissues was tested in detached-leaf assays in grow chambers, and for reproductive structures the study was conducted in greenhouse with infestations at early (flowering) and mid reproductive (mid grain filling) stages. Based on the feeding behavior of the species tested, they were cast in four groups: a) *Anticarsia gemmatalis* and *Chrysodeixis includens*, defoliating only the RR variety with the lowest consumption of foliar area; b) *Spodoptera eridania*, defoliating both RR and IPRO varieties, consuming twice than the species mentioned above; c) *Helicoverpa armigera*, defoliating and being the most damaging species to pods in the RR variety; and d) *S*. *cosmioides* and *S*. *frugiperda*, defoliating and damaging pods in both varieties. The species differed in their ability to feed on IPRO varieties, so a different economic threshold should be considered. Consequently, in cases where more than one species are found simultaneously, the species composition should be considered in estimating the economic threshold. Additionally, our findings may contribute to a better decision-making to control insect feeding injury in IPRO varieties, because a slower larval growth provides more time to ensure the need of control with insecticides. In summary, this clasification contributes to an improved recommendation of sustainable insecticide use, taking into account the behavior of each species that are major soybeans pests in South America.

## Introduction

Soybean is one of the most important row crops globally, with 350 million t harvested per season as of 2019, from which South American countries (mostly Brazil, Argentina, Paraguay, and Uruguay) contribute to nearly 50% of the global production [1]. Insect pests are one of the most important factors limiting soybean productivity in South America. Indeed, the lepidopteran species *Anticarsia gemmatalis*, *Chrysodeixis includens*, *Helicoverpa armigera*, *Spodoptera cosmioides*, *S*. *eridania* and *S*. *frugiperda*, are considered important the most important soybean pests based on their high prevalence and their impact on yield [2–6], and they often occur in a complex of two or more species, and a mix of larval instars.

Currently, management of the lepidopteran species relies mostly on two pillars: use of Bt soybeans and application of foliar insecticides. In particular, Bt soybeans (containing the event MON87701, which expresses the Cry1Ac protein from *Bacillus thuringiensis*) are currently the main component to control feeding injury of lepidopterans pests: *A*. *gemmatalis*, *C*. *includens* and *H*. *armigera* [7–9], and to reduce feeding injury of *Spodoptera* species [10–12]. For *Spodoptera* spp., control might require insecticide applications if their populations are large enough to cause economic injury. In addition, refuge areas may also need to be applied if insect pressure becomes high [13]. However, foliar insecticides need to be applied in a environmentally and economically acceptable way, compatible with IPM (Integrated Pest Management) principles [8, 14, 15]. According to this principle, insecticides should be applied only when insect population size exceeds the economic threshold (ET, population size that causes an economic damage to the crop, higher than the cost of controlling the insects), which considers the time taken to implement control measurements before the feeding injury reaches the economic injury level (EIL), or feeding injury severity that will cause economic loss to the crop [16].

The first step to estimate the EIL is to detemine the consumption of different pest species [17]. In this sense, some work has been done in the past with major soybean pests, which usually did not compare hosts or species [18–21] or studied the consumption of different host plants by the same insect species [22–31]. Also, other studies have focused on soybeans [32, 33], or compared Bt and non-Bt soybeans in a reduced number of species [9, 10, 34, 35]. The broadest characterizations that we are aware of focused on small larvae feeding on vegetative tissues on non-Bt soybeans [36–38] or compared to Bt soybeans [7, 12]. However, since most of the species mentioned above also consume soybean reproductive tissues [2, 3, 32, 39–41], Bt soybeans are widely prevalent in South America, and usually larvae of late stages move from weeds or neighbor crops [41] prompted us to integrate all these aspects in a single report. For this reason, this work aimed to characterize and compare the consumption of small- and medium-size larvae of *Anticarsia gemmatalis*, *Chrysodeixis includens*, *Helicoverpa armigera*, *Spodoptera cosmioides*, *S*. *eridania*, and *S*. *frugiperda* feeding on vegetative and reproductive tissues of Bt and non-Bt soybeans, for a more precise characterization of EIL on soybean.

## Material and methods

### Insects and plants

Insect colonies of *Anticarsia gemmatalis*, *Chrysodeixis includens*, *Helicoverpa armigera*, *Spodoptera cosmioides*, *Spodoptera eridania* and *Spodoptera frugiperda* were obtained from field-collected insects from different areas of Brazil (collecting around 200 insects per location and species) on non-Bt soybean crops, and maintained in the laboratory in controlled conditions (25°C, RH of 70%, photoperiod of 14:10 [L:D] h). Colonies incorporated new insects annually to reduce inbreeding. *H*. *armigera* taxonomic determination was confirmed according to Arneodo et al. [42].

The Bt soybean variety M7739IPRO (containing the event MON87701, which expresses the Cry1Ac protein that confers control of some lepidopteran species at various stages, hereafter referred to as IPRO) and the nearly isogenic (non Bt, hereafter referred to as RR) variety BMX Desafio RR were used. Plants of both varieties were grown in 12-liter plastic pots in greenhouse at room temperature (20–25°C) until the desired growth stage. Plants were watered and fertilized periodically to maximize plant growth.

### Larval consumption at vegetative stages

Soybean leaflets were excised daily from plants at around V6-V8 [43] from the middle third of the plants, cleaned for 2 minutes in 5% Clorox, rinsed with water, and dried with paper towels before placing them in 10-cm diameter Petri dishes (one leaflet per dish). Two larvae of the desired growth stage were then placed in the Petri dishes using paint brushes. Larvae of L1 and L3 growth stages were tested. Water was provided by placing small wet cotton bolls in each Petri dish to avoid dehydration. This experiment was run in a growth chamber with the same settings for insect colony rearing.

After the infestation, the following activities took place daily on each Petri dish: determination of insect survival, measurement of foliar area consumed as percentage of total leaf area [44], and replacement of leaflets if area consumed has exceeded 50%. This procedure continued until all larvae had pupated or died. In cases where no consumption was detected after a two-day period, leaflets were replaced anyway to avoid potential effects of dehydration upon foliar area consumption. Pupal weight (mg) was measured individually.

The defoliation percentage was converted to area (square centimeters) by measuring the area of 20 representative leaflets per variety. These measurements were performed by taking pictures of leaflets placed beside a paper sheet containing a square of known area. The area of each leaflet was then measured in ImageJ [45] using the square for calibration.

### Larval consumption at reproductive stages

Each pot was placed on a tray, and was irrigated by adding water to the trays, avoiding the potential effect of high moisture upon larval mortality. At either early (R2) or late (R5) [43] reproductive growth stages, plants were infested with two larvae of of a given species at the desired growth stage (L1 or L3) using paint brushes. Each plant was covered individually in voile to avoid larval movement between plants.

Fifteen days after the infestation, foliar area consumed, number of injured and uninjured pods and grain yield (g) were measured per plant basis. The foliar area consumed was estimated as the difference between the average foliar area of uninfested plants (ten plants per soybean variety) and the remaining foliar area of each infested plant. Both areas were estimated by taking photographs of leaflets beside a square of known area as described above.

### Experiment design and statistical analyses

Each Petri dish (for vegetative stage) or pot (for reproductive stage) was considered a replication. The number of replications was 30 for vegetative stages and 15 for reproductive stages. Petri dishes and pots were arranged randomly in the growth chamber and greenhouse. The total number of treatments [24] consisted of the interaction of three factors: six insect species, two soybean varieties (IPRO and RR), and two larval growth stages (L1 and L3).

Statistical analyses were performed using Infostat [46]. Predicted values were compared using DGC test [47], with a significance level of 10% for all variables.

Insect survival was analyzed according to Kaplan-Meier for the whole set of treatments. In cases where the p value was below 0.05, the most divergent treatment was determined visually and excluded from the following analysis. This process was repeated until p value exceeded the value of 0.05, indicating that survival of the remaining treatments was similar.

The number of pupae and the proportion of injured pods were analyzed using generalized and mixed linear models. A binary distribution (pupated or not) with a logit linkage function were used for the number of pupae, being insect species, larval size and soybean variety considered fixed effects. Results were expressed as the proportion of larvae reaching pupal stage out of the 30 larvae infesting soybean leaflets. A binomial distribution was used for the number of injured pods, adjusting the proportion using the total number of pods per pot.

Foliar area consumed, pupal weight, proportion of injured pods and grain weight were analyzed using generalized linear models, being insect species, larval size and soybean variety considered fixed effects. Foliar area consumed was also analyzed using a linear regression.

## Results

### Survival of larvae

**Fig 1:**
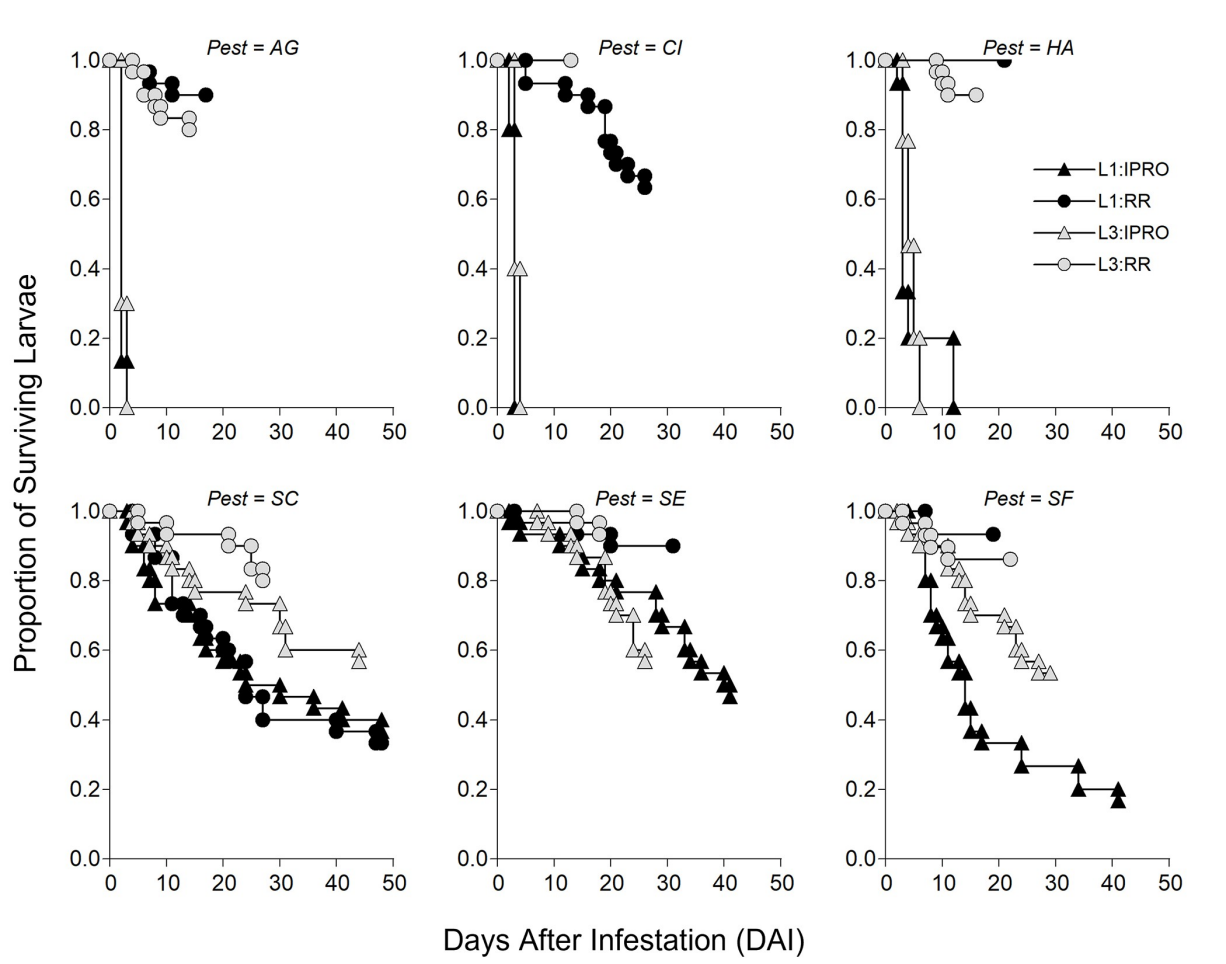
Proportion of surviving larvae over time (days) infesting leaf discs of IPRO (Bt) or RR (non-Bt) soybean varieties with small (L1) or large (L3) larvae. Insect species: AG: *Anticarsia gemmatalis*, CI:, *Chrysodeixis includens*, HA: *Helicoverpa armigera*, SC: *Spodoptera cosmioides*, SE: *S*. *eridania*, SF: *S*. *frugiperda*.

Larval survival (Fig 1) varied across species. In *A*. *gemmatalis* (AG), the initial analysis indicated differences among Stage x Variety combinations (p<0.0001), due to a lower survival in IPRO. When the analysis was repeated in each variety, there was no difference in survival between L1 and L3 in IPRO (p = 0.5171) and in RR (p = 0.2881). In *C*. *includens* (CI) the same initial pattern was seen between varieties (p<0.0001), but L3 survived more than L1 in both IPRO (p = 0.0251) and RR (p = 0.0003). In *H*. *armigera* (HA) the initial difference (p<0.0001) was due to soybean varieties, while in the split analysis there were no differences between stages in IPRO (p = 0.6807), and weakly in RR (p = 0.0781), in this case due to no mortality in L1.

The pattern of survival in *Spodoptera* species was more complex than in the other species tested. In *S*. *cosmioides* (SC) the initial analysis showed significance (p = 0.0013), being the main difference between larval stages, having L1 lower survival than L3. The analysis split by Stage indicated no differences in survival between varieties in L1 (p = 0.3859), and a somewhat significant (p = 0.0610) lower survival in IPRO by L3. In *S*. *eridania* (SE) the first analysis indicated a lower survival by larvae feeding IPRO. The split analysis per variety indicated no effect of larval stage in IPRO (p = 0.6615) and RR (p = 0.6382). Lastly, in *S*. *frugiperda* (SF) there was also a lower survival in larvae feeding in IPRO than in RR but, while there were no differences in survival in RR (p = 0.3742), L3 survived more than L1 in IPRO (p = 0.0037).

### Proportion of pupae

**Fig 2:**
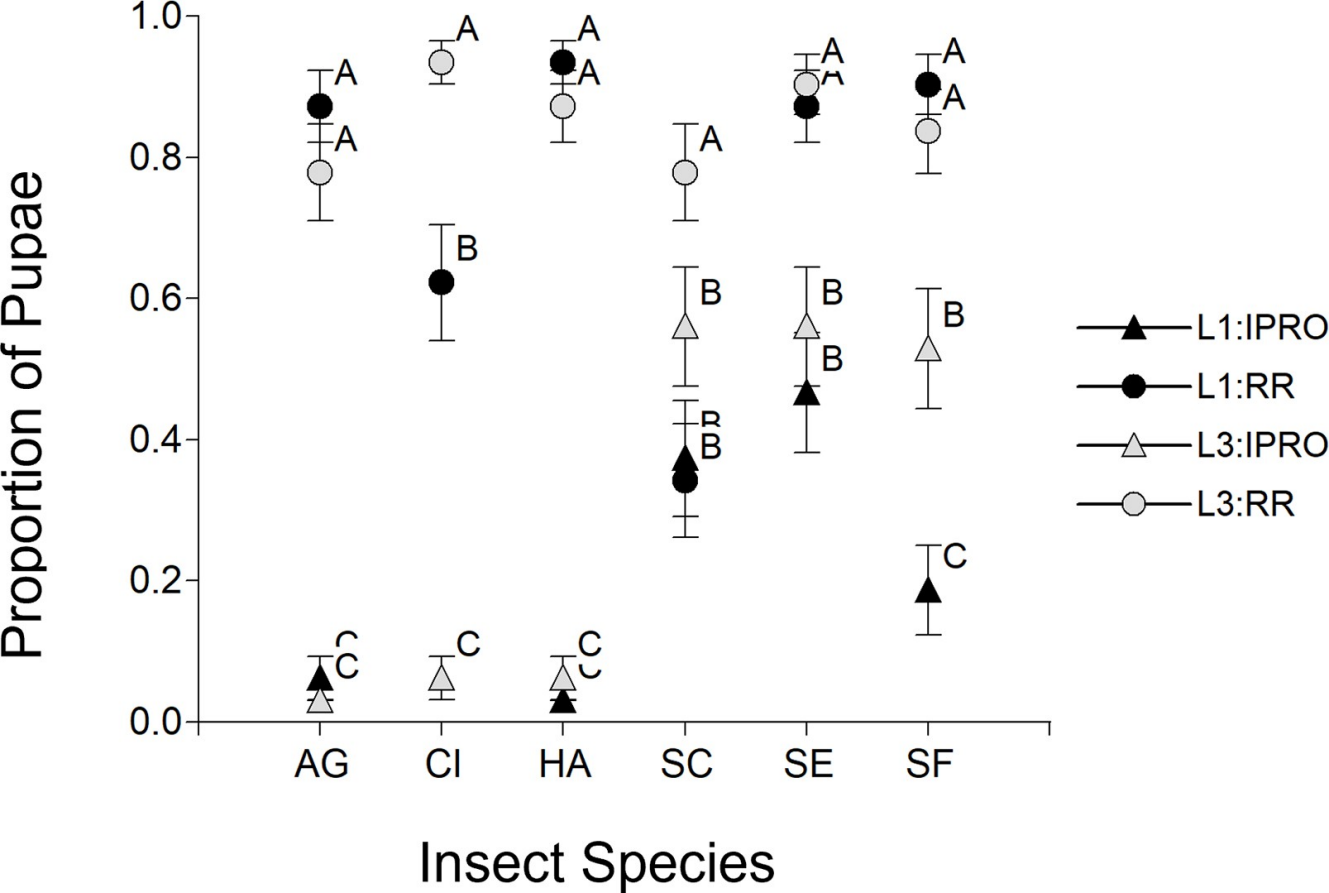
Proportion of larvae reaching pupal stage infesting leaf discs of IPRO (Bt) or RR (non-Bt) soybean varieties with small (L1) or large (L3) larvae. Insect species: AG: *Anticarsia gemmatalis*, CI:, *Chrysodeixis includens*, HA: *Helicoverpa armigera*, SC: *Spodoptera cosmioides*, SE: *S*. *eridania*, SF: *S*. *frugiperda*. Values with the same letter are not significantly different according to contrasts in the mixed model test (α = 0.05). Bars indicate standard error of the mean.

The proportion of larvae reaching pupal stage had a significant effect of Species (p = 0.0018), and the interactions Species x Variety (p<0.0001) and a weak interaction (p = 0.0708) of Species x Stage x Variety. The single effects Stage (p = 0.9971) and Variety (p = 0.9615), as well as the interactions Stage x Variety (p = 0.9998) and Species x Stage (p = 0.6192) were not significant. Fig 2 describes the triple interaction, showing that the Species effect and the Species x Variety interaction were because no larvae of *H*. *armigera*, *A*. *gemmatalis* or *C*. *includens* reached pupal stage, while the species of *Spodoptera* did so. In this sense, all three *Spodoptera* species differed in this aspect: in *S*. *cosmioides* L3 feeding on RR had the highest proportion of pupae compared to the other ones, *S*. *eridania* survived more in RR than in IPRO without effect of larval size, and *S*. *frugiperda* had a higher proportion of pupae in RR than in IPRO, with L3 surviving more than L1 in this latter variety.

### Duration of larval period

**Fig 3:**
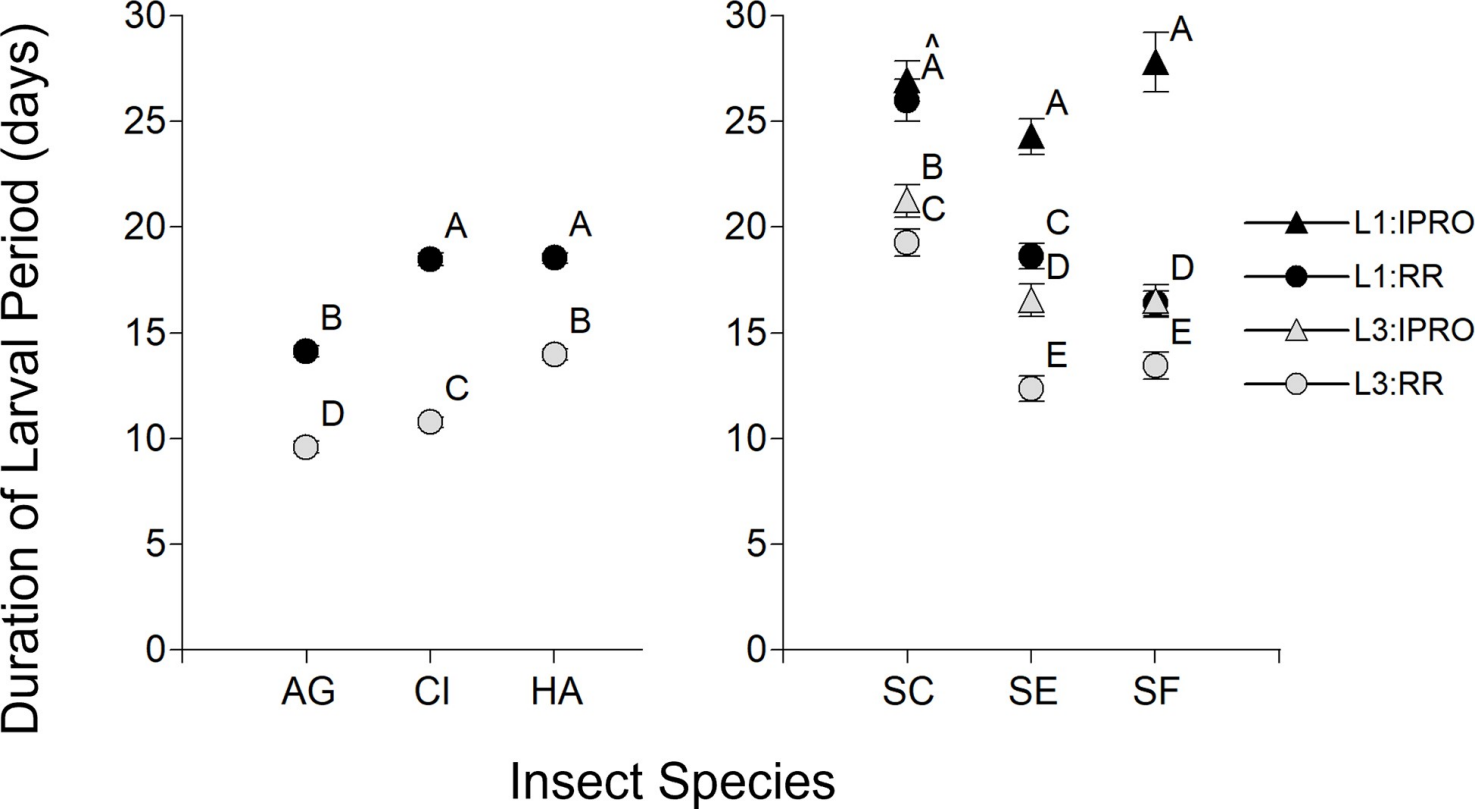
Duration of larval period (days) of larvae infesting leaf discs of IPRO (Bt) or RR (non-Bt) soybean varieties with small (L1) or large (L3) larvae. Insect species: AG: *Anticarsia gemmatalis*, CI:, *Chrysodeixis includens*, HA: *Helicoverpa armigera*, SC: *Spodoptera cosmioides*, SE: *S*. *eridania*, SF: *S*. *frugiperda*. Values with the same letter are not significantly different according to contrasts in the mixed model test (α = 0.05). Bars indicate standard error of the mean.

Because no larvae of *A*. *gemmatalis*, *C*. *includens* and *H*. *armigera* developing on the IPRO variety reached pupal stage (Fig 2), the duration of larval period was analyzed in two parts: a) these three species were analyzed to understand the effect of Stage and Species and their interaction, and b) the *Spodoptera* species were analyzed to see the effect of Stage, Species, Variety and their interaction. In the first comparison (Fig 3 Left), there was a significant effect of Species, Stage, and their interaction (p<0.0001 in all the cases). The Species effect was because of a longer larval period of *H*. *armigera*, and then by *C*. *includens*, and the interaction to the relatively longer length of larval period in L1 of *C*. *includens*. The analysis for *Spodoptera* species showed signifficant efects of Pest, Stage, Variety and Pest x Variety (p<0.0001 in all the cases) and Stage x Variety (p = 0.0025) and for Pest x Stage x Variety (p = 0.0006). The Variety effect was due to a longer duration of larval period in IPRO than in RR variety, and the triple interaction was because this delay differed across species. In *S*. *cosmioides*, the increase in duration of larval period was observed only on L3, in *S*. *eridania* in the same amount in both larval sizes, and in *S*. *frugiperda* mostly in L1.

### Foliar area consumption

**Fig 4:**
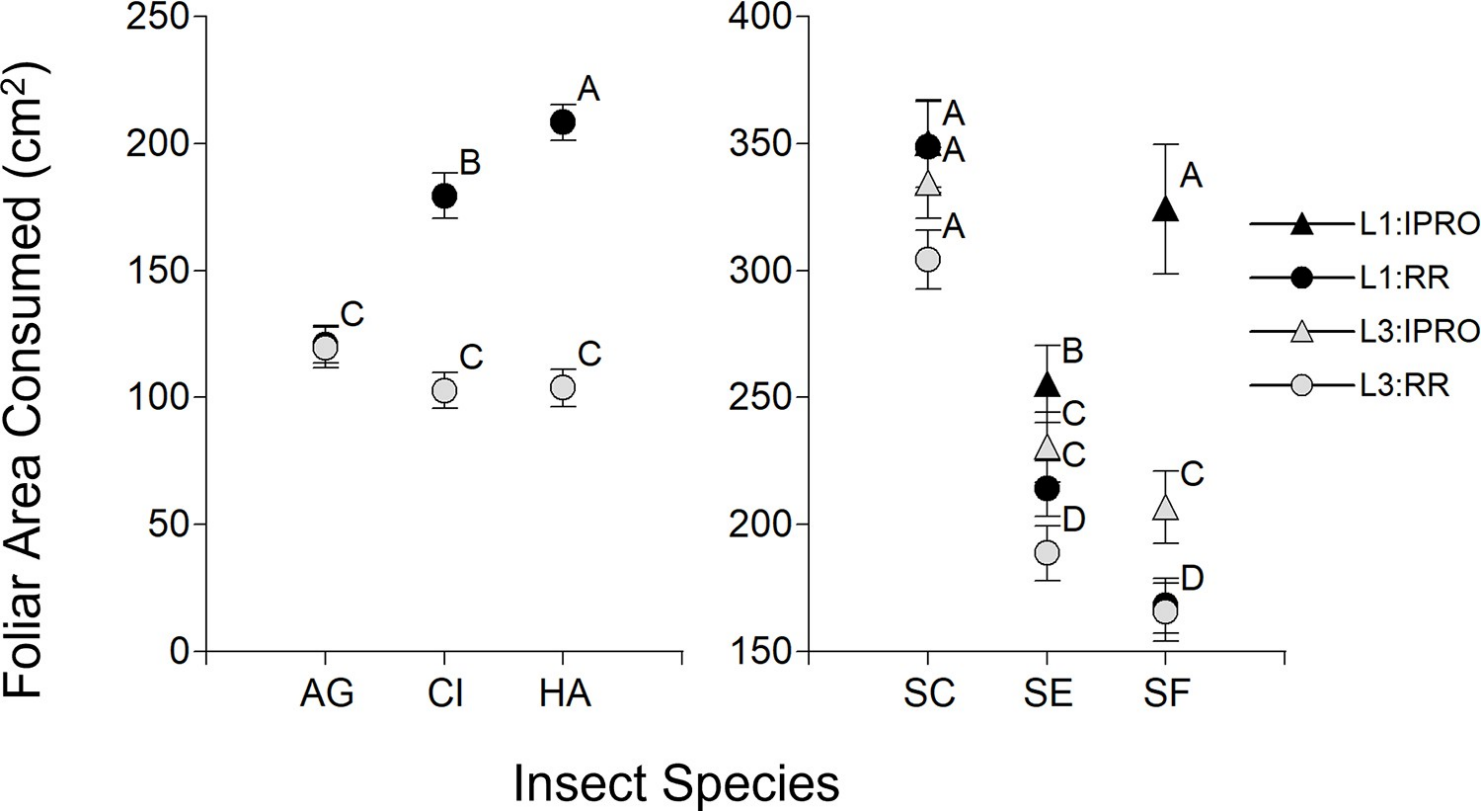
Foliar area consumed (cm^2^) of larvae infesting leaf discs of IPRO (Bt) or RR (non-Bt) soybean varieties with small (L1) or large (L3) larvae. Only larvae that reached pupal stage were included. Insect species: AG: *Anticarsia gemmatalis*, CI:, *Chrysodeixis includens*, HA: *Helicoverpa armigera*, SC: *Spodoptera cosmioides*, SE: *S*. *eridania*, SF: *S*. *frugiperda*. Values with the same letter are not significantly different according to contrasts in the mixed model test (α = 0.05). Bars indicate standard error of the mean.

The foliar area consumed was first analyzed like the duration of larval period, considering only the larvae that reached pupation. In the first comparison (Fig 4 Left), there was a significant effect of Stage, Species, and their interaction (p<0.0001 in all the cases). The Species effect and the interaction with Stage were due mostly to a lower consumption of L1 in *A*. *gemmatalis*. In the *Spodoptera* species (Fig 4 Right), there was significance of single effects Stage, Species, and Variety (p<0.0001 in the three cases), as well as the Species x Variety (p = 0.0011) and Stage x Species x Variety (p = 0.0039), but not from Stage x Variety (p = 0.1009) nor Stage x Species (p = 0.2284) interactions. The triple interaction shows that the foliar area consumed by L1 and L3 on IPRO and RR soybeans varied across species. Indeed, *S*. *cosmioides* had the highest consumption of all the species across larval stages and varieties, *S*. *eridania* consumed less foliar area overall, with a trend of higher consumption in IPRO, and *S*. *frugiperda* consumed the least of the *Spodoptera* spp., with the exception of the high leaf area consumed by L1 on IPRO.

**Fig 5:**
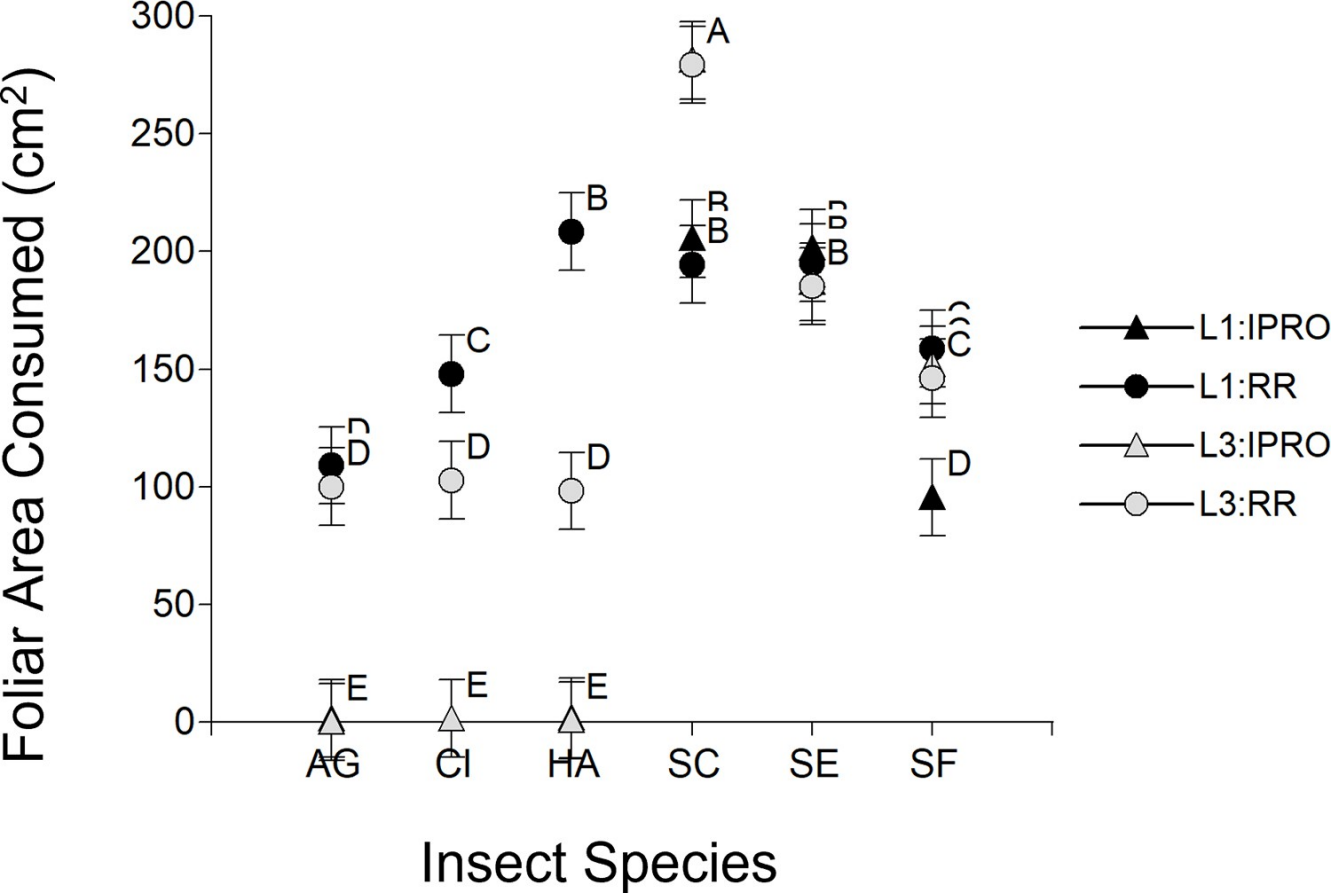
Foliar area consumed (cm^2^) of larvae infesting leaf discs of IPRO (Bt) or RR (non-Bt) soybean varieties with small (L1) or large (L3) larvae. Both larvae that did reach to pupal stage and and those that did not were included. Insect species: AG: *Anticarsia gemmatalis*, CI:, *Chrysodeixis includens*, HA: *Helicoverpa armigera*, SC: *Spodoptera cosmioides*, SE: *S*. *eridania*, SF: *S*. *frugiperda*. Values with the same letter are not significantly different according to contrasts in the mixed model test (α = 0.05). Bars indicate standard error of the mean.

A second approach to analyze foliar area consumed included also larvae that did not reach pupal stage (Fig 5). This analysis indicated significance of Species, Variety, Stage x Species, Variety x Species (p<0.0001 in the cases), Stage x Variety (p = 0.0077) and Stage x Species x Variety (p = 0.0745), but not from Stage (p = 0.9249). The analysis of the triple interaction showed the same pattern for the species that did not survive on IPRO variety (*A*. *gemmatalis*, *C*. *includens* and *H*. *armigera*), because larvae hardly consumed any area before being controlled. On the contrary, the pattern of foliar area consumed changed in all of the *Spodoptera* species. Indeed, in *S*. *cosmioides* L3 consumed more foliar area than L1, in *S*. *eridania* the foliar area consumed was similar in both larval sizes, and in *S*. *frugiperda* L1 in IPRO had the lowest consumption.

**Fig 6:**
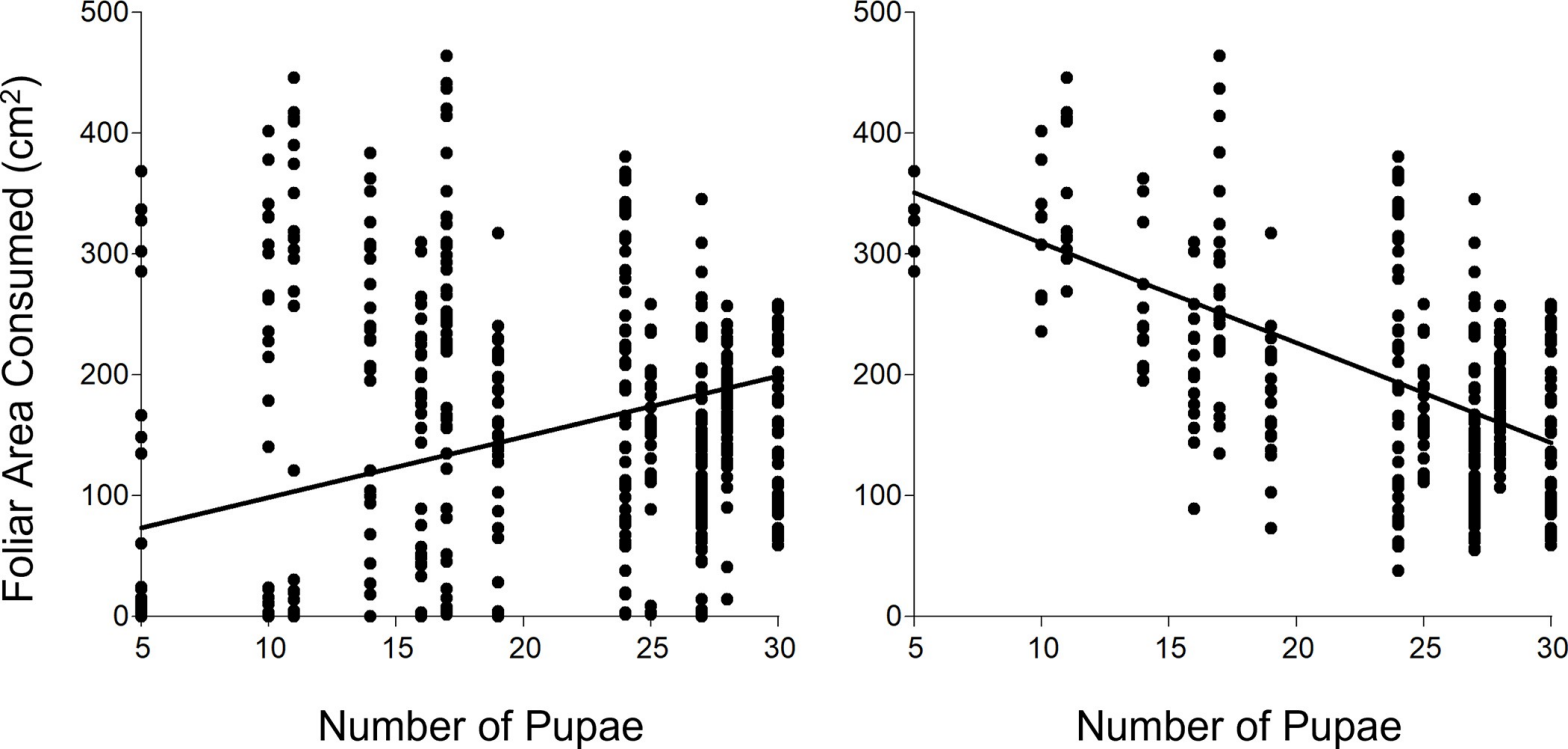
Regression of foliar area consumed (cm^2^) and number of pupae. Left: including larvae that reached pupal stage and those that did not. Right: only larvae that reached pupal stage. All insect species, larval stages at infestation and soybean varieties combined.

The shifts in the pattern of foliar area consumption by L1 and L3 of *Spodoptera* species feeding on IPRO or RR varieties was related to the proportion of larvae reaching to pupal stage (survival). The strongest example were L1 of *S*. *frugiperda* on IPRO, which consumed the lowest or the highest foliar area if all the larvae or only the surviving ones were considered respectively in the analysis. This was related directly to the high mortality of L1 on IPRO (Fig 2). This same shift was also seen in *S*. *eridania*, in which the lower survival in IPRO compensated the higher area consumed by surviving larvae compared to RR, yielding a similar foliar area consumed in both varieties if all the larvae were considered, and also in *S*. *cosmioides*, comparing the consumption of L1 and L3. In this sense, Fig 6 shows that the foliar area consumed increases (5.03 cm^2^ per pupa, p<0.0001, R^2^ 0.20) with the number of pupae when all the larvae were considered (Left), but decreases if only surviving larvae (Right) are considered (-8.28 cm^2^ per pupa, p<0.0001, R^2^ 0.32).

### Pupal weight

**Fig 7:**
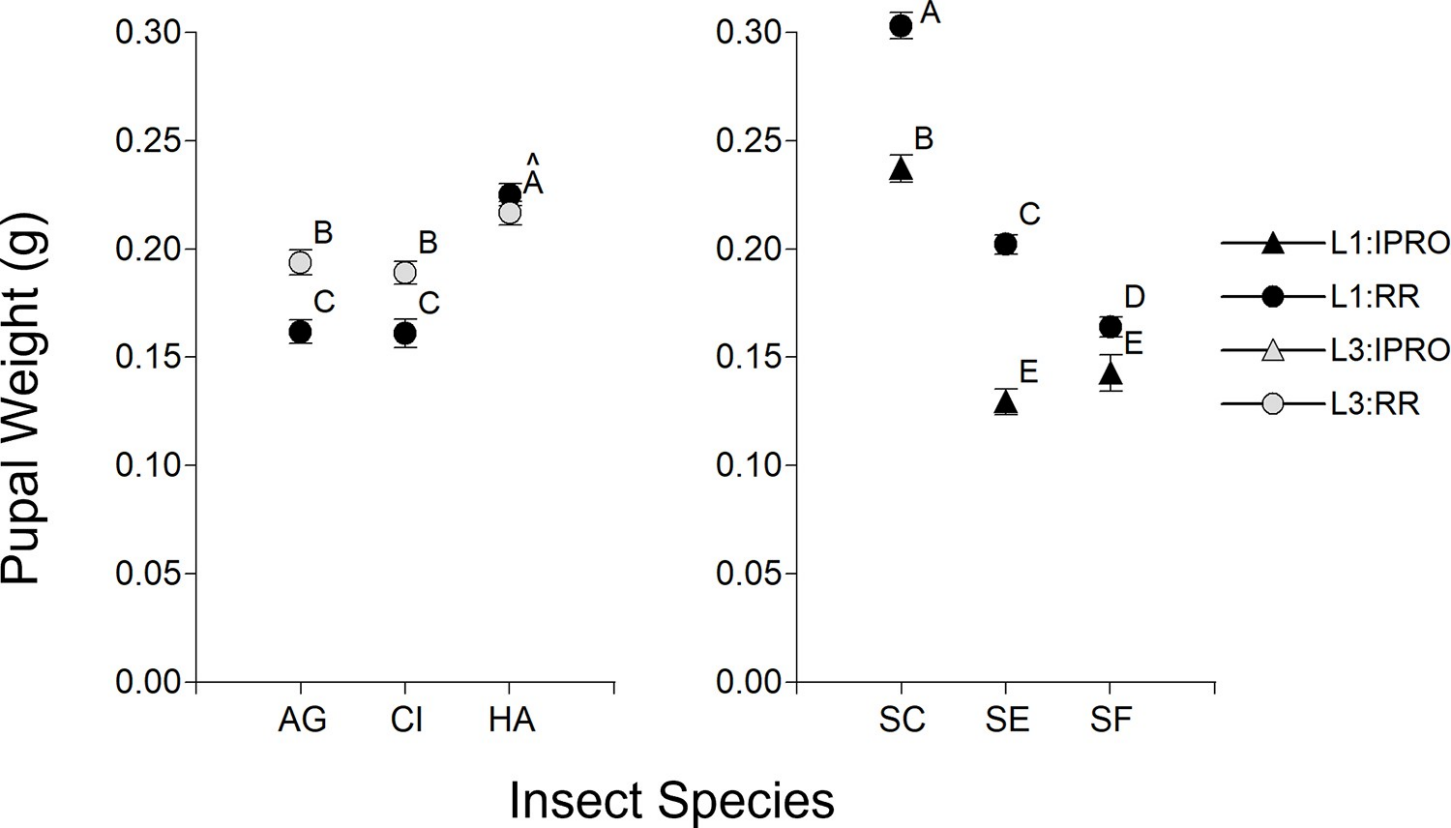
Pupal weight (g) of larvae infesting leaf discs of IPRO (Bt) or RR (non-Bt) soybean varieties with small (L1) or large (L3) larvae. Insect species: AG: *Anticarsia gemmatalis*, CI:, *Chrysodeixis includens*, HA: *Helicoverpa armigera*, SC: *Spodoptera cosmioides*, SE: *S*. *eridania*, SF: *S*. *frugiperda*. Values with the same letter are not significantly different according to contrasts in the mixed model test (α = 0.05). Bars indicate standard error of the mean.

Pupal weight was analyzed like the duration of larval period. In the first comparison (Fig 7 Left), there was a significant effect of Species (p<0.0001), Stage (p = 0.0003), and their interaction (p = 0.0004). The Species effect was due to a larger pupal weight of *H*. *armigera*, and the interaction to a larger pupal weight in L3 than in L1 in *A*. *gemmatalis* and *C*. *includens* but not in *H*. *armigera*. The analysis of the *Spodoptera* species showed a significant effect of Species (p<0.0001), Stage (p = 0.0069), and Variety (p<0.0001), and the interactions Species x Variety (p<0.0001) and Stage x Variety (p = 0.0143), while the interactions Stage x Species (p = 0.4932) and Stage x Species x Variety (p = 0.5021) were not significant. The Species effect was because pupae of *S*. *cosmioides* weighted more than of the other species, and the Species x Variety interaction was due to a largest weight of pupae in RR than in IPRO in *S*. *cosmoides* and *S*. *eridania*, and to a less extent in *S*. *frugiperda* in both L1 (Fig 7 Right) and L3 (not shown). The Stage effect and Stage x Variety interaction were due to larger pupal weight in L3 than in L1 in *S*. *frugiperda* only.

### Larval consumption at reproductive stages

**Fig 8:**
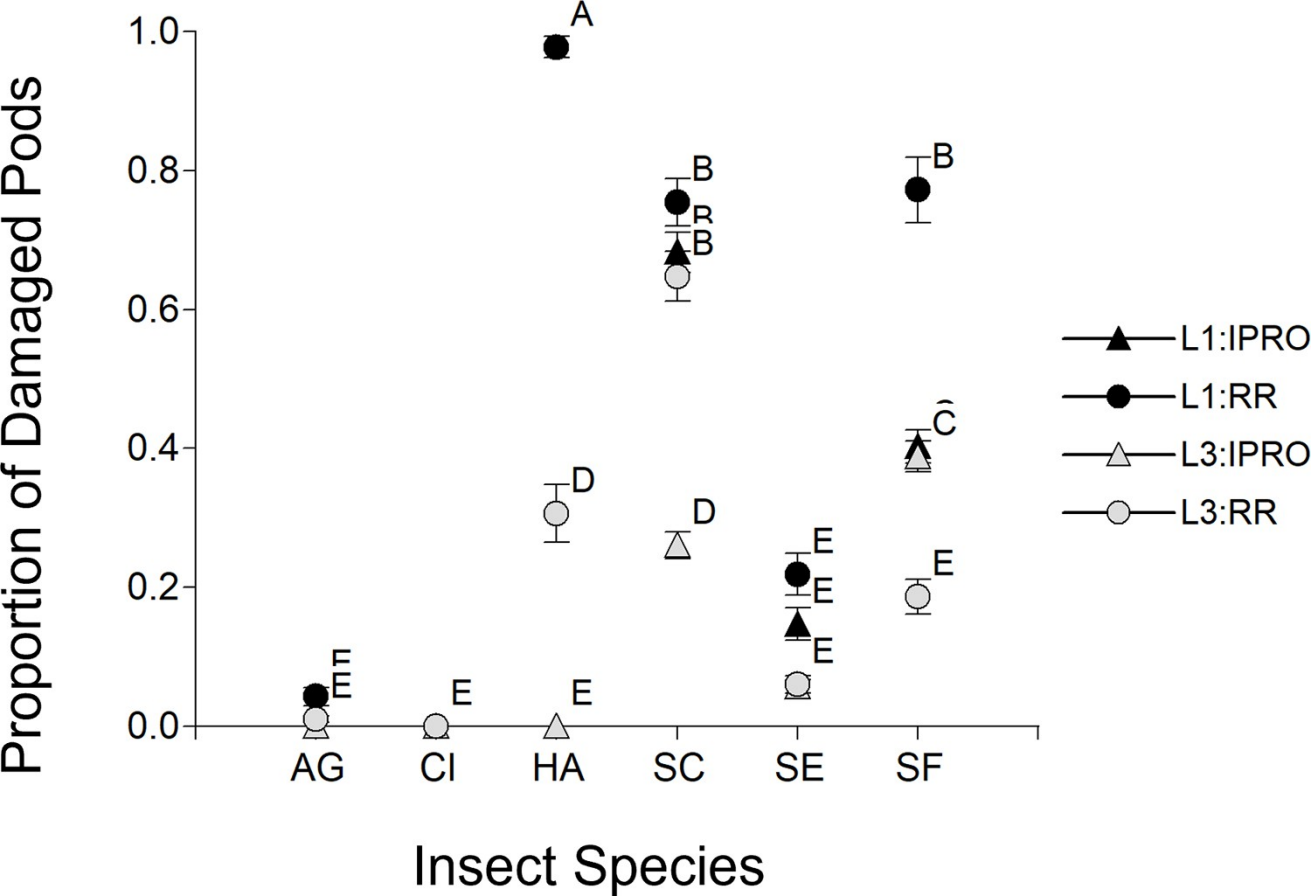
Proportion of injured pods infesting plants of IPRO (Bt) or RR (non-Bt) soybean varieties with small (L1) or large (L3) larvae at medium reproductive stages. Insect species: AG: *Anticarsia gemmatalis*, CI:, *Chrysodeixis includens*, HA: *Helicoverpa armigera*, SC: *Spodoptera cosmioides*, SE: *S*. *eridania*, SF: *S*. *frugiperda*. Values with the same letter are not significantly different according to contrasts in the mixed model test (α = 0.05). Bars indicate standard error of the mean.

The proportion of injured pods with infestation at flowering showed no significant effect of any factor or interaction (results not shown), with less than 1% of injured pods. On the contrary, when the infestation took place at mid grain-filling (Fig 8) there was significance of Species (p<0.0001), Stage (p = 0.0395) and Variety (p = 0.0008) single factors, and of Species x Variety and Species x Stage x Variety (p<0.0001 in both cases) interactions. These significances were grouped into three clusters according to the species: a first group including *A*. *gemmatalis*, *C*. *includens* and partially *S*. *eridania*, where none or very few injured pods were found; a second group composed of *H*. *armigera* where pods were injured only on RR variety, mostly in L1; and a third group consisting of *S*. *cosmioides* and *S*. *frugiperda*, where pods were injured on both IPRO and RR varieties. These two latter *Spodoptera* species injured more pods with infestation at L1, and L3 of *S*. *cosmioides* injured more pods on IPRO, and the opposite taking place in *S*. *frugiperda*. The analysis of regression of grain weight with the number of injured pods (results not shown) showed a negative relationship (0.33 g per injured pod, p<0.0001, R^2^ 0.77).

**Fig 9:**
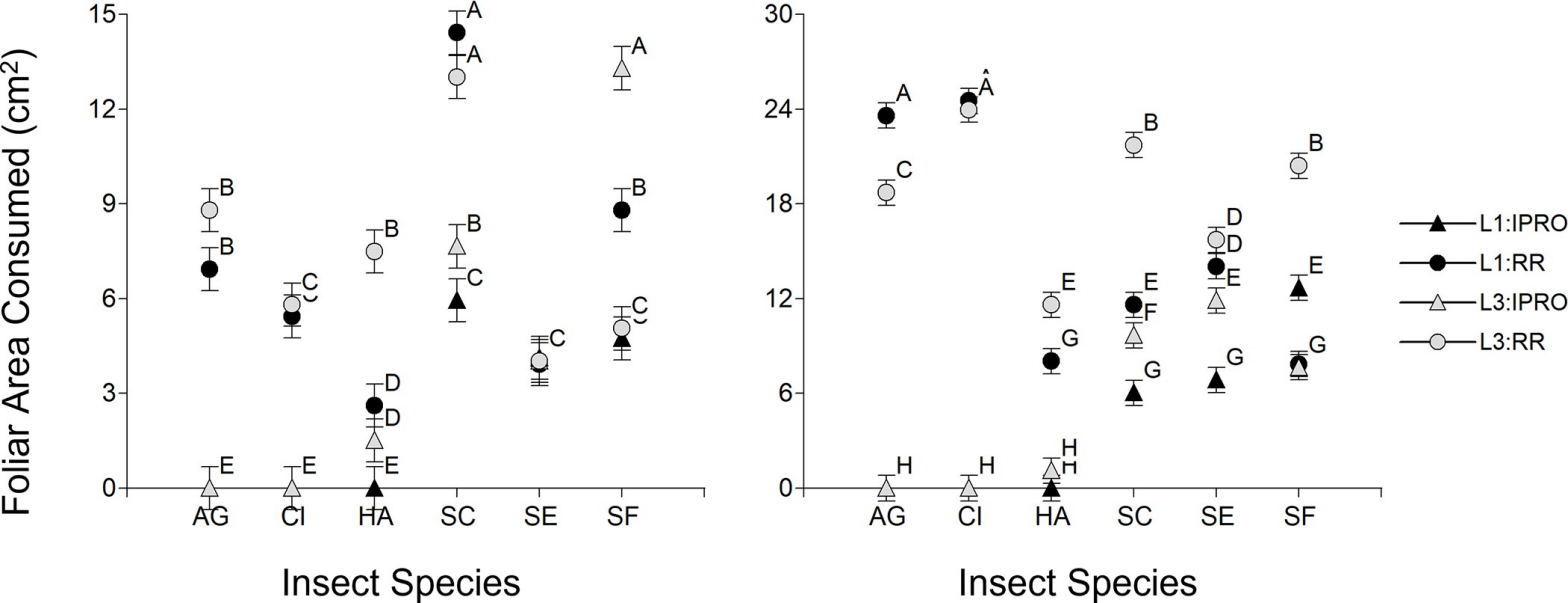
Foliar area consumed (cm^2^) at early (Left) or medium (Right) reproductive stages infesting plants of IPRO (Bt) or RR (non-Bt) soybean varieties with small (L1) or large (L3) larvae. Insect species: AG: *Anticarsia gemmatalis*, CI:, *Chrysodeixis includens*, HA: *Helicoverpa armigera*, SC: *Spodoptera cosmioides*, SE: *S*. *eridania*, SF: *S*. *frugiperda*. Values with the same letter are not significantly different according to contrasts in the mixed model test (α = 0.05). Bars indicate standard error of the mean.

The foliar area consumed with infestation at flowering (Fig 9 Left) showed a highly significant effect of Species, Stage, Variety, Species x Variety and Species x Stage x Variety (p<0.0001 in all the cases), as well as a Stage x Species (p = 0.0019) and Stage x Variety (p = 0.0042). The analysis of the triple interaction showed in that no foliar area was consumed in IPRO variety by *A*. *gemmatalis*, *C*. *includens* nor *H*. *armigera* (this latter one showing some foliar area consumed by L3 in IPRO). The *Spodoptera* species behaved differently, with *S*. *cosmioides* consuming less in IPRO, *S*. *eridania* showing no differences, and *S*. *frugiperda* L1 consumed more in RR and L3 in IPRO. This pattern was also seen with infestation at mid reproductive stages (p<0.0001 of all single factors and their interactions). The only major differences were the higher foliar area consumed by *S*. *cosmioides* in L3 in RR soybeans, a lower consumption in L1 in IPRO soybeans in *S*. *eridania*, and in the pattern of consumption by *S*. *frugiperda* (Fig 9, Right). The analysis of regression of foliar area consumed with the number of injured pods (results not shown) showed a positive relationship (0.21 g per injured pod, p<0.0001, R^2^ 0.26).

## Discussion

Integrating the consumption of foliar area adjusted by survival and proportion of injured pods to obtain an EIL for each species, we identified the following behaviors: a) *A*. *gemmatalis* and *C*. *includens*, causing only defoliation, limited to the RR variety, having the lowest consumption of foliar area; b) *S*. *eridania*, causing majorly defoliation in both RR and IPRO varieties, consuming twice as much foliar area than the abovementioned species; c) *H*. *armigera*, limited to the RR variety causing an intermediate consumption of foliar area and the highest injury to pods; d) *S*. *cosmioides* and *S*. *frugiperda*, causing the highest (*S*. *cosmioides*) or intermediate (*S*. *frugiperda*) injury in both defoliation and to pods in both varieties. Despite having a similar range of feeding, these two latter species differed quantitatively in the ability to feed from IPRO variety, with *S*. *cosmioides* causing similar injury to both varieties and *S*. *frugiperda* showing a trend of lower injury to IPRO. This clasification contributes to a sustainable recommendation of insecticide use, taking into account the behavior of these species that are major soybeans pests in South America.

Survival of lepidopteran larvae was high in all the species tested for the soybean varieties evaluated in this study, reaching up to 90% for some species of L3 larvae. Our results were similar to previous reports for *C*. *includens* [20, 22, 33], *H*. *armigera* [30], *S*. *eridania* [3, 21, 40], and *S*. *frugiperda* [26, 31]. The lower survival of *S*. *cosmioides* was previously reported [23, 32], although some findings using artificial diet [29] indicated higher survival. Survival of larvae up to the pupal stage was affected in three ways: larval size at infestation, soybean variety, and their interaction. Changes in survival due to larval size were seen as higher survival by L3 than L1 in *C*. *includens* and *S*. *cosmioides*. This could be related to differences in the dynamics of survival of these species, or due to an effect of the change from feeding in artificial diet (where they fed until the infestation) to the foliar discs as found before [18]. Changes in survival attribuited to soybean variety were related to the high efficacy of the IPRO biotechnology to control *A*. *gemmatalis*, *C*. *includens* and *H*. *armigera* larvae [7–9, 41], while *Spodoptera* spp. larvae were partially (*S*. *eridania* and *S*. *frugiperda*) or not (*S*. *cosmioides*) controlled, as it was formerly reported [10–12, 24, 35]. The interaction of larval size and soybean variety, changed the survival of *S*. *frugiperda*, expressed as a lower survival of L1 in IPRO than in RR variety, while no differences were seen in L3. To our knowledge, this finding has not been documented yet and could be related to a higher tolerance of large larvae to Bt proteins [10] than to small larvae. This fact may deserve further consideration in the future, since larval survival plays a role in foliar area consumed, and consequently to determine EIL [17].

Length of larval period was similar to previous studies conducted at similar temperatures [20, 22, 23, 29, 35], and ranged from 10 to 35 days according to species, soybean variety and larval size. Besides the obvious effect of larval size at the time of infestation, there were differences among the species tested, with *A*. *gemmatalis* the shortest, and the *Spodoptera* species the longest duration. Larval period in *Spodoptera* species lasted longer in IPRO variety, mainly in small larvae of *S*. *eridania* and *S*. *frugiperda* as reported before [11], while *S*. *cosmioides* was less affected, in agreement with previous work on this species [35]. This finding may contribute to a better decision-making to control foliar pests in IPRO varieties, because a slower larval growth provides more time to decide the need of control with insecticides.

The range of foliar area consumed found in this work is similar to earlier reports for *A*. *gemmatalis* [37], *C*. *includens* [18], *S*. *cosmioides* [37], *S*. *eridania* [3] and *S*. *frugiperda* [37], including larvae that pupated and those that did not. These results confirm that *S*. *cosmioides* consumes about twice the foliar area than the other species, and so must be considered separately to determine economic thresholds. Soybean variety also played a major role on foliar area consumption, since IPRO variety reduced almost entirely the consumption by *A*. *gemmatalis*, *C*. *includens* and *H*. *armigera*. However, foliar consumption was high in the *Spodoptera* species as previously reported [11, 12, 35].

Pupal weight was similar to previous results for the species studied [3, 10, 22, 25, 31]. Pupae of *Spodoptera* spp. on IPRO weighed less than for the RR variety, with a smaller difference in *S*. *cosmioides*; this contradicted previous reports that showed similar pupal weight in both varieties by these *Spodoptera* species [10, 35]. The lower pupal weight found in this study was related to a longer larval period in IPRO, although the foliar area consumed was similar in both varieties. For this reason, we consider that this matter deserves a deeper analysis in the future, since it would help to better understand the fitness of these species on IPRO soybeans.

In the present study, when infestation took place at flowering (R1-R2), there were no injured pods with the insect pressure used (2 larvae per plant). This could be due to the large size of the plants and the high number of flowers at the time of infestation compared to the insect pressure, taking into account that most flowers naturally abort [48, 49]. In turn, the proportion of injured pods during mid-grain filling varied primarily with the feeding habit of each species. On one hand, there were no (*A*. *gemmatalis* and *C*. *includens*) or few (*S*. *eridania*) injured pods in species not considered consistently as pod feeders. An important aspect of this study is that no injury of reproductive structures by *A*. *gemmatalis* was observed. This agrees with the most usual behavior of this species, although other reports [19] showed that this species feeds on soybean pods at R3-R4 stages. Further research on this area may be useful, due to the high prevalence of this species in South America. On the other hand, *H*. *armigera*, *S*. *cosmioides* and *S*. *frugiperda* did injure pods, which is in agreeement with previous results [34] of their feeding behavior. The injury to pods resembled the one to foliar area consumed in *H*. *armigera* damaging RR variety only, and *S*. *cosmioides* and *S*. *frugiperda* damaging both varieties. The results for *H*. *armigera* confirm previous work [7], establishing that species controlled by IPRO during vegetative stages do not injure reproductive structures in the larval sizes and insect pressure used in this study. In turn, while no major differences were seen in the injury to pods by *S*. *cosmioides* for larval size and variety, in *S*. *frugiperda* injury to pods was higher in RR than in IPRO in both larval sizes. These results resemble those of foliar area consumed, and agree with previous work indicating higher tolerance of *S*. *cosmioides* than *S*. *frugiperda* [10, 12, 35] to IPRO varieties.

Another important aspect of this study is that it can help to develop a multi-pest economic injury level (EIL), and hence economic threshold (ET), for the consumption of vegetative and reproductive structures by species able to feed on both varieties. In this work, the presence of reproductive structures did not consistently change the foliar area consumption across species, larval sizes and varieties. For instance, *S*. *cosmioides* was the species with the highest foliar area consumption during vegetative stages, but during reproductive stages this species reduced the consumption of foliar area in favor to feeding from pods. On the contrary, in *H*. *armigera* and *S*. *frugiperda* this shift was seen only on L1 of in RR variety. This is important because, although soybeans tolerates defoliation [37, 38, 50], the aforementioned species do consume pods, reducing yield directly. In this sense, future areas of research could focus in further characterizing this behavior across species.

## Supporting information

S1 File (ZIP)
